# Developing and Evaluating Medical Humanities Problem-Based Learning Classes Facilitated by the Teaching Assistants Majored in the Liberal Arts: A Longitudinal Crossover Study

**DOI:** 10.1097/MD.0000000000002765

**Published:** 2016-02-12

**Authors:** Fen-Yu Tseng, Jeng-Yi Shieh, Tze-Wah Kao, Chau-Chung Wu, Tzong-Shinn Chu, Yen-Yuan Chen

**Affiliations:** From the Department of Internal Medicine (F-YT, T-WK), National Taiwan University Hospital; Department of Physical Medicine and Rehabilitation (J-YS), National Taiwan University Hospital; Graduate Institute of Medical Education & Bioethics (C-CW, T-SC, and Y-YC), National Taiwan University College of Medicine.

## Abstract

Although medical humanities courses taught by teachers from nonmedical backgrounds are not unusual now, few studies have compared the outcome of medical humanities courses facilitated by physicians to that by teaching assistants majored in the liberal arts. The objectives of this study were to (1) analyze the satisfaction of medical students with medical humanities problem-based learning (PBL) classes facilitated by nonmedical teaching assistants (TAF) majored in the liberal arts, and those facilitated by the attending physicians (APF) and (2) examine the satisfaction of medical students with clinical medicine-related and clinical medicine-unrelated medical humanities PBL classes.

A total of 123 medical students, randomly assigned to 16 groups, participated in this study. There were 16 classes in the course: 8 of them were TAF classes; and the others were APF classes. Each week, each group rotated from 1 subject of the 16 subjects of PBL to another subject. All of the 16 groups went through all the 16 subjects in the 2013 spring semester. We examined the medical students’ satisfaction with each class, based on a rating score collected after each class was completed, using a scale from 0 (the lowest satisfaction) to 100 (the highest satisfaction). We also conducted multivariate linear regression analysis to examine the association between the independent variables and the students’ satisfaction.

Medical students were more satisfied with the TAF (91.35 ± 7.75) medical humanities PBL classes than APF (90.40 ± 8.42) medical humanities PBL classes (*P* = 0.01). Moreover, medical students were more satisfied with the clinical medicine-unrelated topics (92.00 ± 7.10) than the clinical medicine-related topics (90.36 ± 7.99) in the medical humanities PBL course (*P* = 0.01).

This medical humanities PBL course, including nonmedical subjects and topics, and nonmedical teaching assistants from the liberal arts as class facilitators, was satisfactory. This pedagogical approach of student-centered, nonmedical topics, nonmedical facilitators, and small groups, which is associated with a deep approach to learning medical humanities, should be highly encouraged.

## Abstract 2

Supplemental Digital Content is available in the text

## INTRODUCTION

“Medical humanities” was defined as “an interdisciplinary field of humanities (literature, philosophy, ethics, history, and religion), social science (anthropology, cultural studies, psychology, sociology), and the arts (literature, theater, film, and visual arts), and their application to medical education and practice.^1^” The term was first mentioned in the article “Medical Humanities—The New Medical Adventure” by an Australian physician who was concerned that the medical syllabus was difficult for students to discuss the broader cultural, philosophical, and societal issues relevant to clinical practice. As such, he worked with his colleagues to design a course structured around classic literacy work.^2^

In 1984, Cassell published an opinion article through the Hastings Center pointing out that the contribution of humanities to medicine is recognized as the shift in medicine from considering the diseases only to seeing a sick person.^3^ In 1993, the General Medical Council in the UK also highlighted the importance of the humanities in medicine by suggesting its integration into curriculum in undergraduate medical education for communication skills, ethical and legal issues relevant to clinical practice, respect for patients and colleagues, and patients’ rights in all respects.^4^

In Taiwan, undergraduate medical education reform started in the 1990s. In 1998, the National Committee on Foreign Medical Education and Accreditation in the US pointed out several drawbacks in Taiwan's undergraduate medical education and also judged Taiwan's undergraduate medical education as noncomparable.^5^ As such, the comments regarding Taiwan's undergraduate medical education further facilitated the birth of Taiwan's Medical Accreditation Council, as well as undergraduate medical education reform, with a particular focus on the integration of the humanities courses to undergraduate medical curriculum.^6^

Medical humanities not only can nurture medical students’ critical thinking, but also their understanding of personal values, empathy, cultural competence, leadership, and teamwork, thus preparing medical students for responding appropriately to complicated clinical problems.^7^ Given that incorporating medical humanities into a medical school's curriculum promotes the development of empathetic, compassionate, and culturally sensitive physicians,^8^ medical humanities are attracting more and more attention in present medical education.^8–10^

Several studies in Taiwan have shown that the outcomes of medical humanities courses are not satisfactory.^11,12^ A study conducted in Taiwan by Tsui and colleagues examined the evolution of medical humanities education using in-depth interviews. For designing a medical humanities curriculum, they suggested borrowing the experiences of scholars who specialized in liberal arts, for example, Chinese literature, Taiwanese literature, history, philosophy, and so on.^13^ Medical humanities courses taught by the scholars specializing in liberal arts rather than in medicine-related fields are not unusual; for example, the University of Missouri-Kansas City School of Medicine in the US reported that some of its medical humanities courses, such as “Healing and Humanities”, “Healing and the Arts”, “Medicine and Law”, and so on, were taught solely by non-MDs.^14^

Although medical humanities courses taught by teachers from nonmedical backgrounds are not unusual now, few studies have examined the outcome of medical humanities problem-based learning (PBL) courses. The objectives of this study were (1) to examine the satisfaction of medical students with medical humanities PBL classes facilitated by the teaching assistants with strong backgrounds in liberal arts, and those facilitated by the attending physicians and (2) to examine the satisfaction of medical students with clinical medicine-related (CMR) and clinical medicine-unrelated (CMU) medical humanities PBL classes.

## MATERIALS AND METHODS

### Medical Humanities Course

A two-credit medical humanities PBL course was required for the second year medical students. The medical students were randomly assigned to 16 groups. There were 16 subjects in the PBL course. Each week, each group rotated from 1 subject of the 16 subjects of PBL to another subject. All of the 16 groups went through all the 16 subjects in the semester (Appendix 1).

The 16 subjects were numbered consecutively from 1 to 16. The 1st, 3rd, 5th, 7th, 9th, 11th, 13th, and 15th subjects were Taiwanese Literature, Traditional Chinese Opera, Medical Ethics, Music, Art History, Western Opera, Art Collections in the Children's Hospital, and English, respectively. Each subject had 1 to 4 different topics from which medical students chose 1 topic for PBL. Please refer to Table 1 for further details about the subjects and topics for the medical humanities PBL course.

**TABLE 1:**
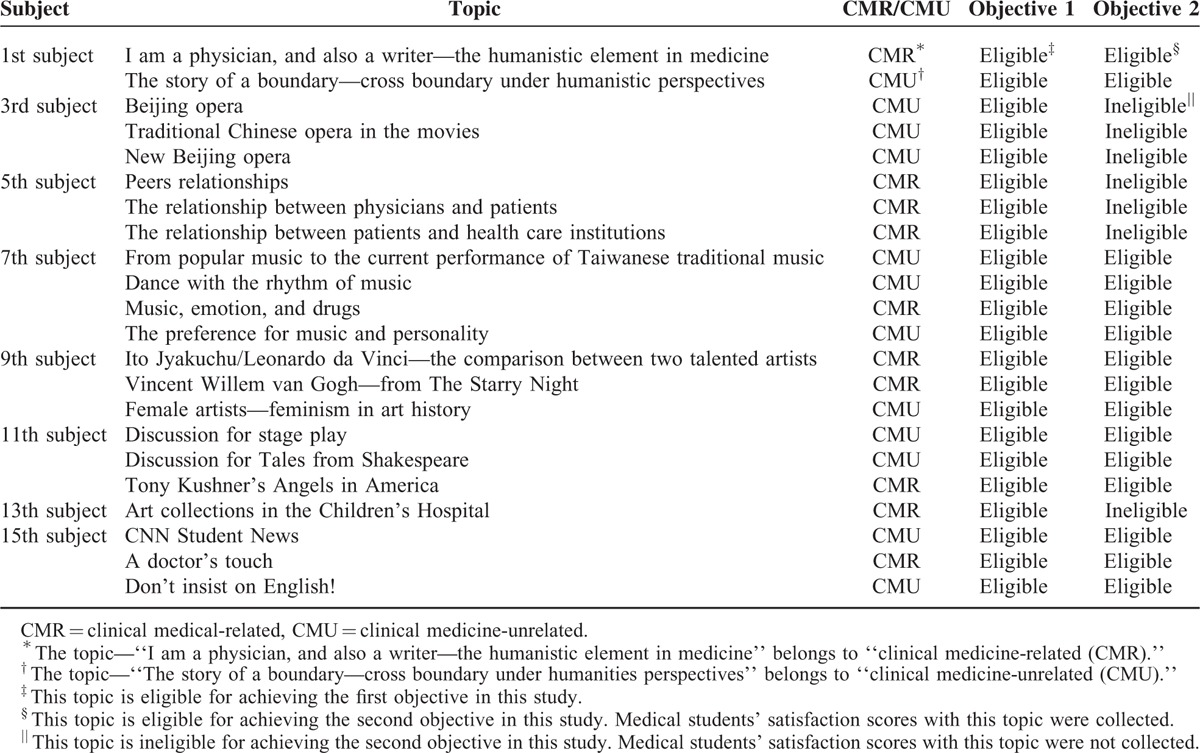
The Subjects and Topics of the Medical Humanities Problem-Based Learning Classes Facilitated by Teaching Assistants With Backgrounds in the Related Fields of Liberal Arts

Each of the odd-numbered subjects of PBL classes was facilitated by a teaching assistant (teaching assistant-facilitated, TAF) who was either a current master's student in a related field in the College of Liberal Arts, or already had a master's degree in a related field from the College of Liberal Arts.

All of the even-numbered subjects of PBL classes were facilitated by an attending physician (attending physician-facilitated, APF). The subject for each PBL class was selected from a group of subjects agreed upon by all the participating attending physicians as medical humanities-related subjects. Those subjects included “Narrative Medicine,” “Modern Medical History in the Western World,” “Diseases and History,” and so on. In addition, the attending physician and the medical students of the same group were also allowed to develop the medical humanities-related subjects which they were interested in for the PBL and conducted the subject they developed in the APF PBL classes.

### Data Collection

The study was approved by the Research Ethics Committee in National Taiwan University Hospital (201302003W). Data for this study were prospectively collected from February 25 to June 10 in 2013. To rate the satisfaction with the PBL, a rating score was collected after each PBL was completed, with a scale from 0 (the lowest satisfaction) to 100 (the highest satisfaction). Therefore, each medical student had a determined satisfaction score with each of the 16 subjects. We also collected the following as independent variables: sex of the medical student, age of the medical student while the PBL was carried out, the means of entering medical school categorized as a writing exam or oral exam, and whether the PBL was facilitated by a teaching assistant with a background in liberal arts.

### Data Analyses

For examining the satisfaction with the TAF medical humanities PBL classes and the APF medical humanities PBL classes, all the satisfaction scores were included in the data analyses. In examining the satisfaction with CMR and CMU medical humanities PBL classes, we excluded the satisfaction scores with the APF medical humanities PBL classes, and also excluded the satisfaction scores of the TAF medical humanities PBL classes that had only 1 topic in the subject.

We used student *t* test to examine the association between a dichotomous variable and a continuous variable, and Chi-square test to examine the association of 2 dichotomous variables. Kolmogorov-Smirnov test was used to test the normality of distribution for the dependent variable—satisfaction score. Multivariate linear regression analysis was conducted to examine the association between the independent variables and the dependent variable. The assumptions of multivariate linear regression models were tested using variance inflation factor (VIF), White test, and Durbin-Watson test for multicollinearity, heteroscedasticity, and autocorrelation, respectively. A *P* ≤ 0.05 was considered as statistically significant. All statistical analyses were conducted using STATA MP 11.0 (StataCorp, College Station, TX) for Windows PC.

## RESULTS

Eight medical students were excluded from this study because they were international students and entered the medical school through a different way other than a writing or oral exam for domestic students. A total of 123 (93.89%) medical students were included for this study. Each group had 7 to 9 medical students.

A total of 1924 (97.76%) satisfaction scores were received. Fort-four satisfaction scores were missing because of unknown reasons (18 missing values), students not attending the class (8 missing values), and cancelled classes (18 missing values). Among the 1924 satisfaction scores, a satisfaction score of 101 was identified by data comparison and was then corrected to 100, with 101 being beyond the maximal value of satisfaction.

Of the 1924 satisfaction scores, 376 (19.54%) were reported by female medical students, 343 (17.83%) were reported by the medical students enrolled by means of an oral exam, and 963 (50.05%) were reported by the medical students in the TAF medical humanities PBL classes. Satisfaction scores reported by the medical students enrolled by means of a writing exam, and in the TAF medical humanities PBL classes were significantly higher than the satisfaction scores reported by those enrolled by means of an oral exam (*P* < 0.01), and by those in the APF medical humanities PBL classes (*P* < 0.01), respectively (Table 2).

**TABLE 2:**
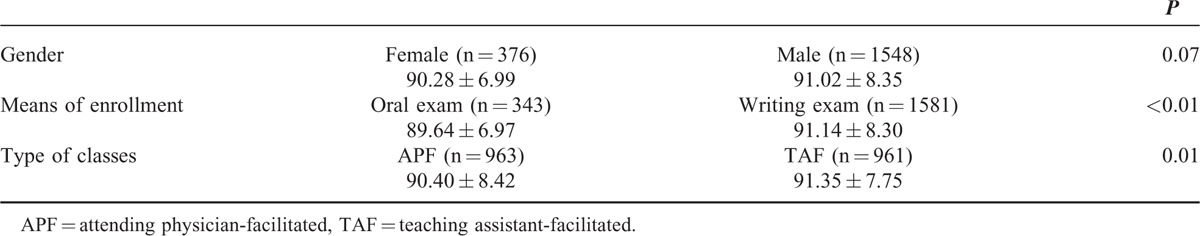
The Comparison of Satisfaction Scores as Stratified by Different Variables

After controlling for other confounding variables using multivariate linear regression, the satisfaction scores with the TAF medical humanities PBL classes were significantly higher by 0.95 based on a scale from 0 to 100 (*P* = 0.01) than those with the APF medical humanities PBL classes (Table 3). The satisfaction scores for this model were distributed normally (*P* = 0.21). All VIFs of each variable were <10, indicating no multicollinearity. Durbin-Watson statistic for the model was 2.011, which was greater than d_u_ 1.715, indicating the residuals from the model were independent. In addition, the errors had a constant variance in the variable (*P* = 0.43), indicating no heteroscedasticity in the model.

**TABLE 3:**
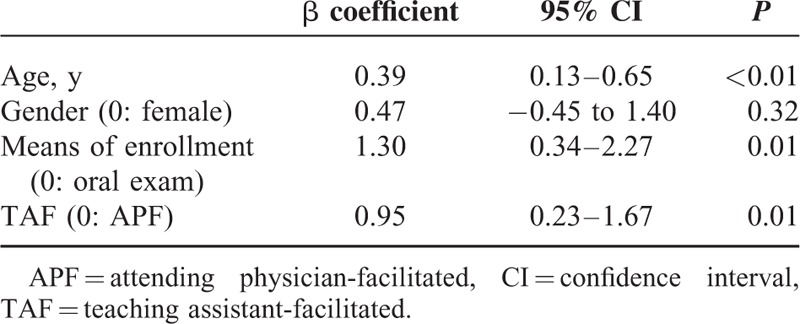
Multivariate Linear Regression Analysis for the Satisfaction Scores With the TAF and APF Medical Humanities Problem-Based Learning Classes

For comparing the satisfaction scores with the CMR medical humanities, PBL classes to those with the CMU medical humanities PBL classes, 963 satisfaction scores reported by the medical students in the APF classes and 360 satisfaction scores reported by the medical students in the 3 TAF classes were excluded because only a single topic was available for the medical students. In the 5 subjects of the TAF PBL classes which in total had 15 topics, 9 (60%) of them were categorized as CMU. A total of 601 satisfaction scores with the 5 subjects were collected. Please see Table 1.

Among the 601 satisfaction scores, 116 (19.30%) were reported by female medical students, 107 (17.80%) were reported by the medical students enrolled by means of an oral exam, and 379 (63.06%) were reported by those in the CMR topics. The satisfaction scores reported by the medical students in the CMR topics were significantly lower than the satisfaction scores reported by those in the CMU topics (*P* < 0.01) (Table 4).

**TABLE 4:**
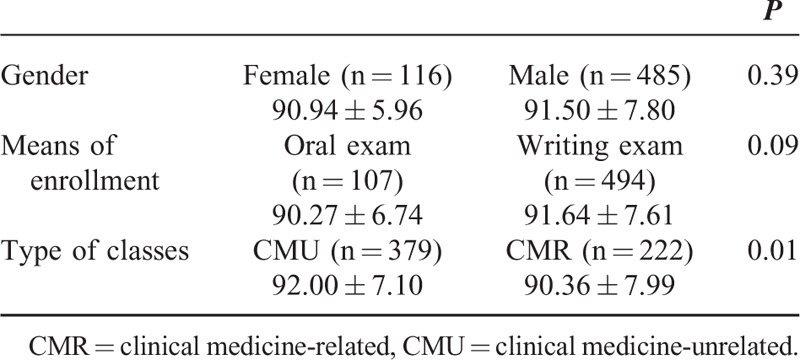
The Comparison of Satisfaction Scores as Stratified by Different Variables

After controlling for other confounding variables using multivariate logistic regression, the satisfaction scores with the CMU topics were significantly higher than those with CMR topics by 1.64 (*P* < 0.01) (Table 5). The satisfaction scores for this model were distributed normally (*P* = 0.21). All VIFs of each variable were <10, indicating no multicollinearity. Durbin-Watson statistic for the model was 1.343, which was less than d_l_ 1.633, indicating the residuals from the model were dependent. In addition, the errors had a constant variance in the variable (*P* = 0.07), indicating no heteroscedasticity in the model.

**TABLE 5:**
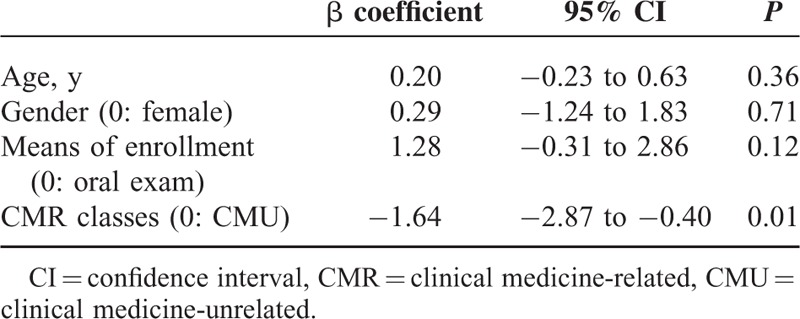
Multivariate Linear Regression Analysis for the Satisfaction Scores With the CMR and CMU Medical Humanities Problem-Based Learning Classes

## DISCUSSION

### Main Outcomes

We organized a new medical humanities PBL course for the second year medical students with 16 subjects in total, with 8 of the subjects facilitated by attending physicians and 8 of the subjects facilitated by teaching assistants with a strong background in the related fields of liberal arts. We found that the medical students were more satisfied with the medical humanities PBL classes facilitated by the teaching assistants having strong backgrounds in the related fields of liberal arts than those facilitated by the attending physicians. We also found that the medical students were more satisfied with the clinical medicine-unrelated topics in the medical humanities PBL course than those of the clinical medicine-related topics.

### The Approaches to Learning Medical Humanities

The number of medical humanities courses have been increasing in medical schools.^15^ However, several studies have pointed out weaknesses of present medical humanities education using lecture, problem-based learning, and bed-side discussion.^16–18^ Wu et al^11^ conducted a qualitative study to examine 11 medical students’ attitudes toward medical humanities courses. They found that medical students were concerned about the inconsistency between learning objectives and courses content, and that medical students preferred more PBL courses than lectures for medical humanities. Kao et al^12^ examined 32 participants’ attitudes toward medical humanities courses. They reported that medical students saw medical humanities courses as useless to their future career because those disciplines were not part of Physicians’ Licensing Exam. Therefore, medical students tended to have a passive attitude toward medical humanities courses according to those studies.

In the 3-P model for student learning proposed by Biggs,^19^ an integrated system composed of 3 main components including Presage, Process, and Product. The Process determines the way a student goes about learning, including the motives for learning and the strategies for engaging in the process of learning to reach the intended outcomes. The combination of motives and strategies forms 3 approaches to learning: (1) a deep approach, for example, understanding meaning and using information; (2) a surface approach, for example, memorizing fact and reproducing information; and (3) an achieving approach, for example, obtaining the highest grades.^20^

PBL has been introduced to medical education by Dr Barrows since the 1960s. This pedagogy was defined by Dr Barrows as (1) student-directed learning; (2) learning done in a small group, ideally 6 to 10 students; (3) a problem that forms the basis for the focus and stimulates learning; (4) the problem is a vehicle for the development of problem-solving skills, and also stimulates the cognitive process; and (5) students in the small group obtain new knowledge through self-directed learning.^21^ In addition, it has been recognized that PBL may potentially promote a deep approach compared with conventional teaching, that is, lecture.^22^ Reid et al^23^ reported that several key factors may encourage a deep approach and discourage surface approach to learning medical humanities: (1) appropriate workload for the learners; (2) clear goals and informative feedback; (3) clear, enthusiastic, empathic teaching focused on promoting conceptual change; (4) freedom of choice over learning content and method; (5) assessment that students perceive to reward understanding; and (6) assessment through written work rather than multiple-choice questions. As such, our medical humanities PBL course, which complied with several of these key factors, was expected to encourage a deep approach to learning medical humanities and to a better learning outcome compared with those taught by conventional teaching, that is, lecture.

### Medical Humanities to Promote Patient-Centered Medical Care

In 1927, Dr Francis Peabody of Harvard Medical School addressed his concern about the lack of humanities training by pointing out “… young graduates have been taught a great deal about the mechanism of disease, …, they are too “scientific” and do not know how to take care of patients.”^24^ Including humanities courses in medical education may potentially provide significant benefits not only to future physicians but also to society as a whole. Those benefits may include enhancing their skills to communicate with patients, family members, and peers,^25^ increasing their ability to observe and recognize diagnostic findings,^26,27^ as well as promoting their empathy and positive attitudes toward psychological aspects of medical encounters.^28^

There is an increasing consensus that medical humanities are good for promoting a patient-centered approach to medical care,^29,30^ which is also highly emphasized in current clinical practice. It is suggested ^28^ that medical students who have been immersed more in humanities are more likely to have positive attitudes toward patient-centered issues and the psychosocial aspects of patient care as indicated by an empathy questionnaire ^31^ and the Attitudes Towards Social Issues in Medicine questionnaire.^32^ As recommended by “Physicians for the Twenty-First Century,” premedical education should be focused on broad and general education including scientific and nonscientific disciplines, not to prepare medical students specifically for medical sciences.^33^ Accordingly, medical humanities courses, as part of the general education for medical students, are mainly conducted in the first and second year for the medical students in National Taiwan University College of Medicine.^34^

No precise definition of medical humanities has been presented in the literature.^35^ Our medical humanities course further extended the boundary of medical humanities by 2 folds: (1) to include nonmedical subjects and topics such as Taiwanese Literature, Traditional Chinese Opera, Music, Art History, Western Opera, Art Collections in the Children's Hospital, and English (Appendix 1) and (2) to invite nonmedical teachers, particularly from liberal arts, to facilitate the PBL classes.

To clearly identify a convincing and widely acceptable indicator to measure the outcome of medical humanities courses is challenging. As reported by Schwartz Wershof et al,^35^ the majority of outcome measures for medical humanities courses in the literature were empathy,^36,37^ professionalism,^38^ and medical students’ self-care.^39^ As indicated by medical students’ satisfaction, the outcome of this innovative course showed that medical students liked nonmedical teaching assistants as facilitators in the PBL classes, as well as the clinical medicine-unrelated topics in the PBL classes.

### Strengths and Limitations

Our study first examined the outcome of the medical humanities PBL course facilitated by the teaching assistants with a strong background in liberal arts as indicated by the medical students’ satisfaction scores. In addition, we also reported that medical students were more satisfied with discussing clinical medicine-unrelated humanities issues rather than clinical medicine-related humanities issues.

Nevertheless, there are some limitations in this study. The first limitation is the generalizability issue. This is a single-center study, and the medical students enrolled in this medical school mostly are ranked in the top 1 percentile on the writing exam, the University Entrance Exam. Therefore, the study results may be limited to be extrapolated to other medical schools.

The second limitation is using the satisfaction scores as the outcome measure to evaluate a new course. Satisfaction with a class only implied the medical students’ perception of the overall class, thus lacking any rich information regarding which element of the class worked well. For example, we did not know whether high satisfaction with a class was attributed to the teaching assistant's endeavor to facilitate the PBL class, or the content of the PBL class. To measure whether medical students’ empathy and professionalism change would be better than to measure satisfaction, however, to end up by proposing that a medical humanities course can promote a medical student's empathy or professionalism is also problematic.

## CONCLUSION

Our medical humanities PBL course, including nonmedical subjects and topics, and nonmedical teaching assistants from liberal arts as class facilitators, was satisfactory. Our study also showed that the extent of the definition of humanities by medical students was broader as indicated by their higher satisfaction with immersing themselves in clinical medicine-unrelated humanities topics. This pedagogical approach of student-centered, nonmedical topics, nonmedical facilitators, and small groups, which may be associated with a deep approach to learning medical humanities, should be highly encouraged. In addition, future studies may be focused on examining various parts of the outcome of medical humanities PBL courses.

## Supplementary Material

Supplemental Digital Content
