# Supplementary material for: Developing and Evaluating Medical Humanities Problem-Based Learning Classes Facilitated by the Teaching Assistants Majored in the Liberal Arts: A Longitudinal Crossover Study
Source: Medicine (Baltimore). 2016 Feb 12;95(6):e2765. doi: 10.1097/MD.0000000000002765 (PMC4753924; doi:10.1097/MD.0000000000002765)
Supplement: Supplemental Digital Content [file medi-95-e2765-s001.doc]

|  | W1 | W2 | W3 | W4 | W5 | W6 | W7 | W8 | W9 | W10 | W11 | W12 | W13 | W14 | W15 | W16 |
| --- | --- | --- | --- | --- | --- | --- | --- | --- | --- | --- | --- | --- | --- | --- | --- | --- |
| G1 | S1 | APF | S15 | APF | S13 | APF | S11 | APF | S9 | APF | S7 | APF | S5 | APF | S3 | APF |
| G2 | APF | S1 | APF | S15 | APF | S13 | APF | S11 | APF | S9 | APF | S7 | APF | S5 | APF | S3 |
| G3 | S3 | APF | S1 | APF | S15 | APF | S13 | APF | S11 | APF | S9 | APF | S7 | APF | S5 | APF |
| G4 | APF | S3 | APF | S1 | APF | S15 | APF | S13 | APF | S11 | APF | S9 | APF | S7 | APF | S5 |
| G5 | S5 | APF | S3 | APF | S1 | APF | S15 | APF | S13 | APF | S11 | APF | S9 | APF | S7 | APF |
| G6 | APF | S5 | APF | S3 | APF | S1 | APF | S15 | APF | S13 | APF | S11 | APF | S9 | APF | S7 |
| G7 | S7 | APF | S5 | APF | S3 | APF | S1 | APF | S15 | APF | S13 | APF | S11 | APF | S9 | APF |
| G8 | APF | S7 | APF | S5 | APF | S3 | APF | S1 | APF | S15 | APF | S13 | APF | S11 | APF | S9 |
| G9 | S9 | APF | S7 | APF | S5 | APF | S3 | APF | S1 | APF | S15 | APF | S13 | APF | S11 | APF |
| G10 | APF | S9 | APF | S7 | APF | S5 | APF | S3 | APF | S1 | APF | S15 | APF | S13 | APF | S11 |
| G11 | S11 | APF | S9 | APF | S7 | APF | S5 | APF | S3 | APF | S1 | APF | S15 | APF | S13 | APF |
| G12 | APF | S11 | APF | S9 | APF | S7 | APF | S5 | APF | S3 | APF | S1 | APF | S15 | APF | S13 |
| G13 | S13 | APF | S11 | APF | S9 | APF | S7 | APF | S5 | APF | S3 | APF | S1 | APF | S15 | APF |
| G14 | APF | S13 | APF | S11 | APF | S9 | APF | S7 | APF | S5 | APF | S3 | APF | S1 | APF | S15 |
| G15 | S15 | APF | S13 | APF | S11 | APF | S9 | APF | S7 | APF | S5 | APF | S3 | APF | S1 | APF |
| G16 | APF | S15 | APF | S13 | APF | S11 | APF | S9 | APF | S7 | APF | S5 | APF | S3 | APF | S1 |

Abbreviation List: G1 ~ G16=group 1 ~ group 16; W1 ~ W16=the first week ~ the sixteenth week; S1=the 1^st^ subject, Taiwanese Literature; S3=the 3^rd^ subject, Traditional Chinese Opera; S5=the 5^th^ subject, Medical Ethics; S7=the 7^th^ subject, Music; S9=the 9^th^ subject, Art History; S11=the 11^th^ subject, Western Opera; S13=the 13^th^ subject, Art Collections in the Children’s Hospital; S15=the 15^th^ subject, English; APF=attending physician-facilitated.

The medical students of group 1 (G1): “Taiwanese Literature” (S1) in the 1^st^ week (W1); the topic for small group discussion facilitated by the attending physician (APF) in the 2^nd^ (W2), 4^th^ (W4), 6^th^ (W6), 8^th^ (W8), 10^th^ (W10), 12^th^ (W12), 14^th^ (W14), and 16^th^ (W16) week; “English” (S15) in the 3^rd^ week (W3); “Art Collections in the Children’s Hospital” (S13) in the 5^th^ week (W5); “Western Opera” (S11) in the 7^th^ week (W7); “Art History” (S9) in the 9^th^ week (W9); “Music” (S7) in the 11^th^ week (W11); “Medical Ethics” (S5) in the 13^th^ week (W13); and “Traditional Chinese Opera” (S3) in the 13^th^ week (W15).
